# Decreased Serum Levels of Interleukin-4 and Interleukin-21 in New Pemphigus Vulgaris Patients, but Not Chronic Patients With Inactive Disease Compared to Healthy Controls

**DOI:** 10.5826/dpc.1102a35

**Published:** 2021-04-12

**Authors:** Pegah Shahbazian, Maryam Izad, Maryam Daneshpazhooh, Hossein Mortazavi, Zahra Salehi, Shirin Behruzifar, Soheil Tavakolpour, Arghavan Azizpour

**Affiliations:** 1 Autoimmune Bullous Diseases Research Center, Department of Dermatology, Razi Hospital, Tehran University of Medical Sciences, Tehran, Iran; 2 Department of Immunology, School of Medicine, Tehran University of Medical Sciences, Tehran, Iran; 3 MS Research Center, Neuroscience Institute, Tehran University of Medical Sciences, Tehran, Iran; 4 Dana-Farber Cancer Institute, Harvard Medical School, Boston, USA

**Keywords:** pemphigus vulgaris, ELISA, cytokine, interleukin-4, interleukin-21

## Abstract

**Background:**

Pemphigus is a rare group of autoimmune blistering diseases with unknown etiology and unclear pathogenesis. Pemphigus vulgaris (PV) is the most common subtype, and is characterized by ulcerations or flaccid blisters on mucous membranes and on the skin. It is accepted that cytokines have a critical role in the pathogenesis of PV, while their exact roles remain to be elucidated.

**Objectives:**

This study assessed serum levels of interleukin (IL)-4 and IL-21 in different phases of the disease in comparison with healthy controls.

**Methods:**

In a case-control cohort design, serum levels of IL-4 and IL-21 were determined by ELISA in three groups: patients with newly diagnosed PV, patients with chronic, inactive PV (PV in remission), and healthy controls.

**Results:**

The study included 88 individuals (58 women and 30 men), including 26 with newly diagnosed PV, 33 with PV in remission, and 29 healthy controls. A significant difference was found among the groups for IL-21 (P = .044), but not for IL-4 (P = .374). Serum levels of IL-4 and IL-21 in newly diagnosed patients were significantly lower than in healthy controls (P = .005 for both), but these cytokine levels in patients with PV in remission were not different from those of controls (P = .343 and P = .221, respectively). Also, no differences in cytokine levels were detected between the newly diagnosed patients and patients with PV in remission. Regardless of disease phase, we detected significantly lower levels of IL-21 in patients than controls (P = .027), but no differences for IL-4 (P = .374).

**Conclusions:**

IL-4 and IL-21 are involved in PV pathogenesis and disease severity. More studies are required to clarify the role of IL-4 and IL-21 in immunopathogenesis and immune response during PV.

## Introduction

Pemphigus is a group of potentially fatal, chronic, autoimmune blistering diseases affecting the skin and mucous membranes. Although the exact pathogenesis of this disease is not clearly understood, it is accepted that autoantibodies against desmoglein 1 (Dsg1) and Dsg3, produced by B cells, are the main cause of disease. In addition to B cells, autoreactive T cells seem to be required in the initial immunological steps for autoantibody induction [1]. Indeed, the function of some T cells is required for the development, function, and antibody production of B cells and plasma cells during pemphigus [2]. In contrast, most regulatory T cells (Tregs) can negatively influence B cell differentiation and function. T cells, which are sources of several cytokines and chemokines, not only affect other immune cells belonging to the innate and adaptive immune systems, but also can act in an autocrine manner. Several cytokines have been studied in pemphigus patients so far [3,4]. Some of these cytokines, such as interleukin (IL)-4, are believed to be pathogenic [5] and some others, such as IL-10, could be a double-edged sword [6]. Regarding the roles of some other newly identified, less studied cytokines, such as IL-21 and IL-35, it is hard to judge, but they are probably pathogenic and protective, respectively [7,8]. In addition to indirect roles of T cells in the pathogenesis of pemphigus, direct recognition of anti-Dsg3 by Dsg3-specific CD4+ T helper cells has been suggested [9–11].

To date, several subsets of T helper (Th) cells have been identified. These subsets, which include Th1, Th2, Th9, Th17, Th22, follicular T helper (Tfh), and Treg subpopulations, could reciprocally regulate each other. Previously, pemphigus used to be known as a Th-2 dominant disease, while increasing pieces of evidence are suggesting a critical role of other subsets, such as Th17 and Tfh [12–14]. As already mentioned, cytokines do not only affect B cells, but could also affect other Th cells. IL-4 and IL-21 are 2 critical cytokines involved in the differentiation of naïve Th cells into Th2 and Th17, respectively. Additionally, IL-21 seems to be essential for Tfh development [7]. To date, different studies have explored the role of IL-4, and the majority of them are consistent with the suggested pathogenic role of this cytokine. IL-4 is mainly produced by Th2 cells and causes Th2 cell differentiation in an autocrine manner. Additionally, it induces isotype switching toward IgG4, a well-recognized class of IgG antibodies involved in pemphigus [15]. IL-21 is another critical cytokine in the immune system that plays a role in the pathogenesis of immune-mediated diseases. It is mainly produced by natural killer T, Th17, and Tfh cells. It leads to the promotion of CD4+ T cells, including Th2, Th17, and Tfh cells; CD8+ cytotoxic T lymphocytes are also affected by IL-21 [7]. It also causes the production of IgG in addition to negatively affecting Tregs.

In this study, we evaluated serum levels of IL-4 and IL-21 in 2 groups of patients with pemphigus vulgaris (PV), including newly diagnosed patients with active disease and patients with chronic, inactive disease, and compared them with healthy controls.

## Materials and Methods

### Study Design and Inclusion Criteria

During a 2-year period (2016–2018), patients with newly diagnosed PV at the Department of Dermatology at Razi Hospital who met the inclusion criteria (see below) were included in the study (newly diagnosed PV group). Additionally, patients with chronic but inactive PV (PV in remission group) as well as a similar number of matched healthy controls were included. Before inclusion, all patients were examined by at least 1 dermatologist, and the diagnosis of PV was confirmed based on clinical features, detectable autoantibodies against Dsg1 or Dsg3, histopathology, and positive perilesional direct immunofluorescence studies.

### Patient Selection and Cytokine Assay

The inclusion criteria for patients were: (1) confirmed diagnosis of PV, (2) treatment-naïve status for new cases and minimal or no therapy for patients with PV in remission (based on the 2008 consensus statement on definitions of pemphigus) [16], and (3) absence of biological therapies in the past 6 months. Patients in the PV in remission group were either in complete remission, defined as having no new or established lesions for at least 2 months, or partial remission, defined as the presence of transient new lesions that heal within 1 week without treatment. Those in the control group were completely healthy and had no history of any skin disease, autoimmune disease, or malignancy.

Patients in remission who developed a new lesion within the past 2 months or who had old, persistent lesion(s) were excluded. Also excluded were pregnant women, patients with other subtypes of pemphigus, and, during the study, any patient in the PV in remission group whose disease became active, defined as the development of new lesions that did not heal within 1 week without additional treatment.

All study subjects provided written informed consent. After signing the consent letter, 6 mL of whole blood was taken from each contributor. Serum was separated and then stored in −70°C for further experiments. Serum levels of IL-4 and IL-21 were measured using ELISA kits (Thermo Fisher Scientific).

### Study Evaluation and Statistical Analysis

Demographic data, history of the disease, and type of therapy used were recorded and collected for each patient. Results for serum concentrations of cytokines are presented as a mean ± standard deviation and median. To compare the mean concentrations in the groups of patients, an independent *t* test was used. To compare more than 2 samples separately, a one-way ANOVA test was used. Statistical analyses were performed using SPSS v.20.0 (IBM Corp., Armonk, NY, USA). P values <.05 were considered significant. Data were graphed using the Graphpad Prism program (version 7.00, GraphPad Software Inc.).

### Ethical Considerations

The study was approved by the Ethics Committee of Tehran University of medical Sciences before initiating, and all persons who were enrolled provided written informed consent. The study was conducted in accordance with the Declaration of Helsinki. Patients’ information was kept confidential and no patient/individual paid any charges.

## Results

A total of 88 patients was enrolled, including 26 (29.5%) newly diagnosed PV patients, 33 (37.5%) patients with PV in remission, and 29 (33%) healthy controls who were age- and sex-matched to the 59 PV patients. In total, 58 (65.9%) were females and 30 (34.1%) were males, with a mean age of 42.2 ± 10.5 years (range, 20–81). Demographic data for each group are shown in Table 1.

**Figure 1:**
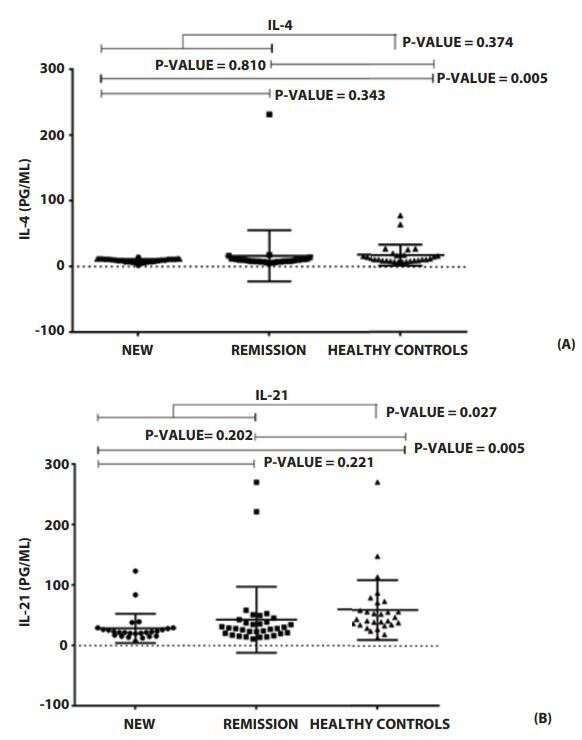
Serum levels of IL-4 (A) and IL-21 (B) in patients with newly diagnosed PV (“New” group), patients with chronic, inactive PV (“Remission” group), and healthy controls.

Table 2 reports the serum levels of IL-4 and IL-21 in the 3 groups, and Figure 1 graphically shows the serum levels of (A) IL-4 and (B) IL-21. Regarding IL-4, no significant difference among the groups was found (P = .374, ANOVA). However, new cases had significantly different serum levels of IL-4 than healthy controls (P = .005). Indeed, new patients with active disease had lower mean levels of IL-4 than healthy individuals. In contrast, the mean of IL-4 in patients with PV in remission was comparable to that of the control group (P = .810). We did not find any significant difference between the 2 groups of PV patients (P = .343). When the 2 groups of patients were combined and considered as a total patients group, no significant difference was noted with healthy controls (P = .374).

Regarding IL-21, based on ANOVA, serum levels of IL-21 were significantly different among the 3 groups (P = .044). We observed a significantly lower mean serum level of IL-21 in new patients (P = .005). However, there was no significant difference between patients with PV in remission and healthy controls (P = .202), as well as between the 2 groups of patients (P = .221). Interestingly, we detected significantly lower levels of IL-21 in total PV patients (both new and remission groups) than in the healthy controls group (P = .027).

## Discussion

This study shows that serum levels of IL-4 and IL-21 are significantly lower among PV patients than healthy controls. Moreover, serum levels of IL-21 could be different between newly diagnosed PV patients and patients with PV in remission. Our results for IL-4 imply a role of IL-4 in the early phase of the disease, but not in a chronic, inactive phase of PV. Previous studies have suggested both increased [17,18] and unchanged [19,20] serum levels of IL-4 in PV patients compared to controls. In contrast with several pieces of evidence implying pathogenic roles of IL-4 [5,21], we observed lower serum levels in new patients than in healthy controls. Regarding IL-21, we again found surprising results that were different from what we expected. Although there is no study on serum levels of IL-21 in PV patients, there is some evidence of the pathogenic role of IL-21 in PV. Yuan et al showed that most T lymphocytes infiltrating PV lesions were CD4+ T helper cells expressing IL-21 [22]. Moreover, not only elevated plasma IL-21 concentrations have been reported in PV patients, but also Dsg3-specific autoreactive T cells producing IL-21 were detected upon ex vivo stimulation with Dsg3 [11]. All this evidence led to suggesting a possible pathogenic role of IL-21 in pemphigus [7]. Additionally, in our recent study [23] on the evaluation of *IL21* gene expression at the transcription level, we did not find any difference between pemphigus patients and healthy controls. However, 3 months after rituximab administration, the expression of *IL21* had increased. These controversies between previous studies and the current study might be due to this study’s limitations or even suggest a high heterogeneity of PV, which could be managed with the view of personalized medicine [23].

Although we did not find any significant differences in the cytokines between the 2 groups of PV patients, different cytokine profiles during the disease phases are plausible. In addition, treatment, previous exposure to certain biologics (eg, rituximab), and duration of disease could change the cytokine profiles of the patients. For example, in our recent study, we showed that, 3 months after rituximab administration, the expression of *EBI3*, *PDCD1*, *IL21* and *IL22* had increased (24]. Additionally, in another study, we showed that the outcome of treatment with rituximab was different between patients treated early or late [25]. This finding might be explained by switching between the immune responses’ arms as time passes or might depend on the exposure to certain therapeutic agents.

In this study, we tried to minimize the effects of treatment by including only patients who were off therapy or on minimal therapy. However, this study still has some limitations, which should be addressed. Firstly, patients in the PV in remission group were not homogeneous. In fact, they were either in complete or incomplete remission. Secondly, the majority of blood samples in the control group were taken from parents or siblings of the patients. Therefore, because of their similar genetic backgrounds, these samples might have introduced some errors in the results and might not be representative of the healthy population. Thirdly, the severity of patients’ disease was not considered. Calculation of PDAI scores, measurement of anti-Dsg1 and anti-Dsg3 levels, and evaluation of their associations with serum cytokines in a study with a larger number of patients are recommended for future studies.

## Conclusions

In conclusion, due to significantly different serum levels of IL-4 and IL-21 among the newly diagnosed PV patients and healthy controls, it can be speculated that these cytokines are involved in the development and pathogenesis of PV. However, according to the literature and due to the mentioned limitations of this study, especially the selection of controls, our results, especially the lower level of these cytokines in patients than in controls, might not be absolutely trustable. Therefore, future studies without the mentioned limitations are required.

## Figures and Tables

**Table 1:** Demographic Data of the Included Patients and Healthy Participants

		Newly Diagnosed PV	PV in Remission	Healthy Controls	Total
Gender	Male, n (%)	13 (43.3)	9 (30.0)	8 (26.7)	30 (100)
	Female, n (%)	13 (22.4)	24 (41.4)	21 (36.2)	58 (100)
Age, mean ± SD (range), y		44.6 ± 9.0 (32–70)	43.6 ± 9.2 (28–59)	38.5 ± 12.4 (20–81)	42.2 ± 10.5 (20–81)

PV = pemphigus vulgaris.

**Table 2:** IL-4 and Il-21 Levels (pg/mL) in Different Phases of the Disease

	Group	No.	Mean ± SD	Median	Range	
IL-4	Newly Diagnosed PV	26	8.63 ± 2.58	9.06	1.88–13.75	.374
	PV in Remission	33	15.92 ± 38.75	8.75	5.0–231.2	
	Total	59	12.71 ± 29.06	8.75	1.88–231.2	
	Healthy Controls	29	17.89 ± 15.99	12.50	7.5–78.13	
IL-21	Newly Diagnosed PV	26	28.21 ± 23.81	21.94	7.1–122.7	.044
	PV in Remission	33	42.35 ± 54.19	28.03	10.84–269.9	
	Total	59	36.13 ± 43.76	25.00	7.1–269.9	
	Healthy Controls	29	40.65 ± 49.37	43.97	13.3–269.9	

IL = interleukin; PV = pemphigus vulgaris.
